# Rethinking health care commercialization: evidence from Malaysia

**DOI:** 10.1186/s12992-015-0131-y

**Published:** 2015-11-19

**Authors:** Vitalis Chukwudi Nwagbara, Rajah Rasiah

**Affiliations:** Institute of Graduate Studies, University of Malaya, 50603 Kuala Lumpur, Malaysia; Faculty of Economics and Administration, University of Malaya, 50603 Kuala Lumpur, Malaysia

**Keywords:** Public health, Performance, Commercialization, Utilization ratios, Hospitals, Malaysia

## Abstract

**Background:**

Against the backdrop of systemic inefficiency in the public health care system and the theoretical claims that markets result in performance and efficiency improvement, developing countries’ governments have been rapidly commercializing health care delivery. This paper seeks to determine whether commercialization through an expansion in private hospitals has led to performance improvements in public hospitals.

**Methods:**

Inpatient utilization records of all public hospitals in Peninsular Malaysia over the period 2006–2010 were used in this study. These records were obtained from the Ministry of Health. The study relied on utilization ratios, bed occupancy rates (BOR), bed turnover rates (BTR) and average length of stay (ALOS). The data were analyzed using SPSS 22 Statistical Software and the Pabon Lasso technique.

**Results:**

Over 60 % of public hospitals in Malaysia are inefficient and perform sub-optimally. Average BOR among the public hospitals was 56 % in 2006 and 61 % in 2010. There was excessive BTR of 65 and 73 times within the period. Overall, the ALOS was low, falling from 3.4 days in 2006 to 3.1 days in 2010.

**Conclusions:**

This study demonstrates that commercialization has not led to performance improvements in the public health care sector in Malaysia. The evidence suggests that efforts to improve performance will require a focus directly on public hospitals.

**Electronic supplementary material:**

The online version of this article (doi:10.1186/s12992-015-0131-y) contains supplementary material, which is available to authorized users.

## Background

Health care provision is recognized as a social responsibility of governments [1, 2]. However, its provision has increasingly been debated because of inefficiency and underperformance of public hospitals in many parts of the world. Spurred by mainstream economic theories which posit that the market will allocate the service efficiently [3], governments of developing countries have started to rapidly commercialize its provision. This has generated a divergence in efficiency by ownership. Burgeoning public health care expenditure has acted as the prime driver of its commercialization.

Arguments about the effects of commercialization on hospital performance can be contextualized on the basis of ownership structure, i.e. public and private. In this setting, three schools, notably, the public choice theory, property rights theory and the principal agent theory stand out. These theories posit that a change in a hospital’s mission, incentive structure and control mechanism will lead to better performance and efficiency [4–8]. The theories suggest that commercial entities perform better and are more efficient than the public ones.

The conceptual analyses of the performance of both sectors have also emerged from the institutional perspective based on ownership structure characteristics. These studies, however, report mixed findings. Berendes and colleagues, analyzed technical quality and responsiveness of ambulatory care services rendered by both ownerships in the developing countries [9]. In their meta-analysis of 80 studies comparing their performances, the authors conclude that in terms of responsiveness, the private sector seems to perform better, but there are no significant differences in technical quality provided by the sectors.

Similarly, Montagu et al. [10] focusing on clinical outcomes, in another meta-analysis identified 21 studies that compared both sectors. There were no statistical differences reported in the meta-analysis of death rates. Indeed the clinical services offered by both sectors are equivalent. A recent review undertaken by Basu and colleagues included the 6 thematic framework outlined in the *World Health Report 2000* [11]. The review identified 59 empirical studies and 13 meta-analyses. They found evidence of higher responsiveness, over-treatments and high patient charges in the private health care sector.

Other studies arrived at similar conclusions. For example, higher rates of unneeded procedures, most especially, cesarean operations, as well as higher prescription drug costs have been widely reported across the private hospital sector [12–19]. Results from countrywide comparative studies are not different either. In South Africa, it was reported that over half of women undergoing antenatal care and giving birth in private health care facilities had to undergo cesarean operations in comparison with less than 20 % in the public sector [13]. Similarly, in China, [20] as well as Malaysia [21], the commercialization of health care has sparked an increase in out-of-pocket spending.

Overall, albeit the evidence on whether private hospitals perform better than public hospitals is inconclusive [22], health care commercialization has continued unabated, especially in the developing countries [23, 24]. In light of the lacuna, there is a need to assess if the increased focus on private hospitals has helped improve the performance and efficiency of public hospitals especially in the developing countries.

Past research on health care performance assessment were carried out using hospital capacity utilization ratios (HCUR) [25–27]. Three ratios constitute the HCUR, namely, BOR, BTR and ALOS [28]. While HCUR offers insights into the overall slack in hospitals, they are fraught with problems [29] as they do not take into account the different hospital categories, such as, public and mission hospitals [30]. Also, past works have used small and non-representative samples.

Hence, we use the entire population of public hospitals in Peninsular Malaysia and differentiate them by their categories. Malaysia is a good laboratory to investigate if the promotion of private hospitals has led to efficiency improvements in public hospitals. The shift towards private health services since the early 1980s, among other reasons, was promoted by the Malaysian government to help reduce burgeoning government expenditure and to reduce the pressure on overcrowded public hospitals [31]. Especially since 1997 the government launched through the Seventh Malaysia Plan health tourism [31], despite the World Bank stressing that markets can fail because of the peculiarity of health care services [32]. The government also began to own private hospitals beginning with the Johor Economic Development Corporation, launching the *Kumpulan Perubatan Johor* (KPJ) hospital chain, which was established in 1981. As at 2014, KPJ had grown in size and coverage, administering 25 commercial specialist hospitals across Malaysia. In addition, the government sold and commercialized the Sabah Medical Centre into Likas Maternity Hospital and Queen Elizabeth General Hospital [33, 34].

As a consequence, the non-commercial share of health care expenditure, which was stable at around 94 % over the period 1977–1981, began to fall gradually from then on to reach 52 % in 1997 as ownership of private hospitals increased sharply thereafter [35]. Health tourism was promoted to offset a fall in demand when the financial crisis struck in the second half of 1997 to reduce demand from Malaysians [36]. Thirty members of the Association of Private Hospitals Malaysia (APHM) were granted health tourism status by the government in 2001. This qualified them for industrial building allowances, tax holidays, and tax rebates for expenses incurred on pre-employment training [31].

The total number of private hospitals classified as health tourist hospitals rose to 41 in 2015 [37]. While Malaysian investors can benefit from tax incentives and capital allowances from the acquisition of high-tech equipment and expansion of domestic clients through claimable medical tax allowance and insurance, the promotion of health tourism was also targeted to attract foreign patients on a large scale. Thus, commercial hospital beds increased from 1171 in 1980 to 10,405 in 2003, which raised the share of commercial ownership of beds from 3.9 % in 1980 to 26.7 % in 2003 [38]. Therefore, the objectives of this study are to analyze the efficiency and performance of public hospitals since the government started promoting commercialization strongly.

## Methods

The study examines the performance of public hospitals using data from all public hospitals in Peninsular Malaysia over the years 2006 till 2010. The data were obtained from the Ministry of Health (MOH), Malaysia, and comprises all hospitals under the auspices of the MOH. The data were subsequently analyzed using Microsoft excel and SPSS 22 statistical software. The study was approved by the Ethics Committee, Ministry of Health, Malaysia (NMRR-08-1581-2976).

**Fig. 1:**
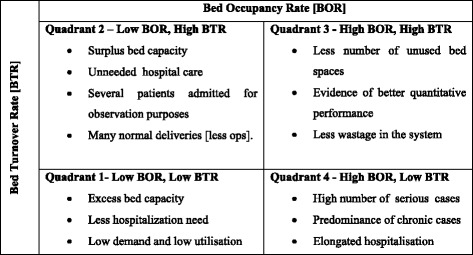
Description of the Pabon Lasso graph

The Pabon Lasso framework is used to analyze public hospital efficiency and performance. This is a graphical method that uses all three utilization measures, namely, BOR, BTR, ALOS simultaneously [39]. The graph (Fig. 1) is divided into four quadrants by horizontal and vertical lines. These lines stand for the means of BOR and BTR, which the corresponding gradient of the lines connecting the beginning point to any point on the chart constitutes the ALOS of the hospital analyzed [30]. However, because the selection of the cutoff levels at the mean values of BOR and BTR could be disputed, Lasso proposed a margin of one standard deviation from the mean of both measures. Also, because this approach has its shortcomings [40], we have included interviews to compliment the results.

Quadrant 1 in Fig. 1 comprises hospitals with low BOR and low BTR, which denote low utilization. Quadrant 2 comprises hospitals with low BOR but high BTR, which shows low utilization but over-capacity. Quadrant 3 is occupied by hospitals with high BOR and BTR, which is ideal from the efficiency viewpoint because it shows high utilization without excess capacity. Quadrant 4 constitutes hospitals with high BOR but low BTR, which shows high utilization but may also show either poor management or bed occupation of long term illness patients. Hospitals in this quadrant are likely to treat long-term diseases, such as, tuberculosis, cancer and psychiatric cases. Based on the assumptions behind the Pabon Lasso technique, only hospitals located in quadrant 3 are efficient.

The hospital performance measures of BOR, BTR, and ALOS were computed based on accepted international working definitions [41] as follows:

i $$ \mathrm{B}\mathrm{O}\mathrm{R} = \frac{\mathrm{Total}\ \mathrm{in}\mathrm{patient}\ \mathrm{days}\ \mathrm{in}\ \mathrm{the}\ \mathrm{given}\ \mathrm{year}\kern0.37em  \times 100}{\mathrm{Total}\ \mathrm{in}\mathrm{patient}\ \mathrm{bed}\ \mathrm{days}\ \mathrm{in}\ \mathrm{the}\ \mathrm{given}\ \mathrm{year}} $$

ii $$ \mathrm{B}\mathrm{T}\mathrm{R}=\frac{\mathrm{Total}\ \mathrm{number}\ \mathrm{of}\ \mathrm{discharges}\ \left[\mathrm{including}\ \mathrm{deaths}\right]\ \mathrm{in}\ \mathrm{the}\ \mathrm{given}\ \mathrm{year}}{\mathrm{Average}\ \mathrm{bed}\ \mathrm{count}\ \mathrm{in}\ \mathrm{the}\ \mathrm{given}\ \mathrm{year}} $$

iii $$ \mathrm{ALOS}=\frac{\mathrm{Total}\ \mathrm{length}\ \mathrm{of}\ \mathrm{stay}\ \mathrm{in}\ \mathrm{the}\ \mathrm{given}\ \mathrm{year}}{\mathrm{Total}\ \mathrm{number}\ \mathrm{of}\ \mathrm{discharges}\ \left[\mathrm{including}\ \mathrm{deaths}\right]\ \mathrm{in}\ \mathrm{the}\ \mathrm{given}\ \mathrm{year}} $$

The following table shows the number of public hospitals, beds occupied, inpatient admissions, bed days, BOR, BTR and ALOS over the period under study.

As shown in Table 1, there were 87 public hospitals in both 2006 and 2010 with 24,361 beds in 2006 and 25,852 beds in 2010. Bed numbers in the hospitals grew on average by 6.1 % per annum over the period 2006–2010. The Available Bed Days (ABD) increased by 16.1 % from 8,891,765 in 2006 to 9,435,980 in 2010. The number of hospitalized patients increased by 17.6 % from 1,564,149 in 2006 to 1,840,044 2010.

**Table 1:** Number of all public hospitals, beds and utilization indicators, Malaysia, 2006 and 2010

	2006	2010
Number of Hospitals	87	87
Total number of active beds	24,361	25,852
Available Bed days [ABD]	8,891,765	9,435,980
Total inpatient admissions	1,564,149	1,842,900
Occupied Bed Days [OBD]	5,239,899	5,694,561
BOR	55.96 %	60.28 %
BTR	65.41 times	74.16 times
ALOS	3.35 days	3.09 days

Computed from inpatient records of all public hospitals in Peninsular Malaysia, 2006 and 2010

## Results

**Fig. 2:**
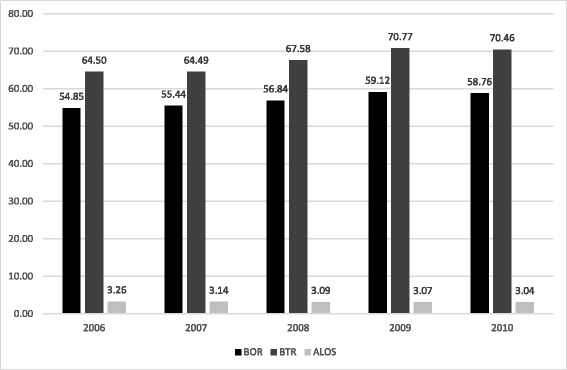
BOR, BTR, ALOS of the public hospitals in Peninsular Malaysia, 2006–2010

The results using the three measures suggest that improvements have taken place in the performance of public hospitals over the period 2006–2010 (see Fig. 2). The BOR increased every year from 54.85 % in 2006 to 59.12 % in 2009 before falling slightly to 58.76 in 2010. By categories of the public hospitals, BOR of general hospitals rose from 77.0 % in 2007 to 78.7 % in 2008 before falling to 76.4 % in 2010 (Table 2). The BOR of specialist district hospitals rose every year from 61.3 % in 2006 to 69.5 % in 2010, while BOR of non-specialist district hospitals rose from 51 % in 2006 to 56.5 % in 2009 before falling to 54.7 % in 2010. While trend improvements in BOR can be seen in all three types of hospitals, they remained significantly lower than the international threshold [42].

**Table 2:** BOR, BTR, ALOS of different public hospital categories, 2006–2010

	2006	2007	2008	2009	2010
BOR					
GH	77.0	77.4	78.7	75.5	76.4
SDH	61.3	62.9	64.3	67.1	69.5
NSDH	51.0	51.1	53.0	56.5	54.7
BTR					
GH	67.5	67.9	68.6	70.6	70.5
SDH	65.6	67.3	71.8	73.9	76.0
NSDH	68.4	67.5	71.5	75.8	74.9
ALOS					
GH	4.2	4.2	4.3	4.0	4.0
SDH	3.4	3.6	3.5	3.6	3.6
NSDH	2.9	2.9	2.8	2.8	2.8

Computed from inpatient records, public hospitals, Peninsular Malaysia, 2006 and 2010

*GH* general hospitals, *SDH* specialist district hospitals, *NSDH* non-specialist district hospitals

Similarly, the BTR increased from 64.5 times in 2006 to 70.8 times in 2009 before falling slightly to 70.5 times in 2010. The BTR of general hospitals rose in trend terms from 67.5 times in 2006 to 70.5 times in 2010 (Table 2). The BTR of specialist district and non-specialist district hospitals rose from 65.6 times in 2006 and 68.4 times respectively in 2006 to 76.0 and 74.9 times respectively in 2010. These figures were significantly higher than the mean BTR of public hospitals in OECD at 44.0 times [43].

ALOS fell every year from 3.3 days in 2006 to 3.0 days in 2010. Among hospitals ALOS of general and non-specialist district hospitals fell in trend terms from 4.2 to 2.9 days respectively in 2006 to 4.0 and 2.8 days respectively in 2010 (Table 2). ALOS of specialist district hospitals rose in trend terms from 3.4 days in 2006 to 3.6 days in 2010. Nevertheless, this figure still fell significantly short of the OECD average of 7.1 days in 2008 [43].

**Fig. 3:**
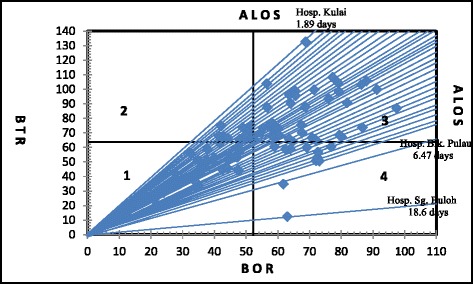
Pabon Lasso graph, public hospitals, Peninsular Malaysia, 2006

**Fig. 4:**
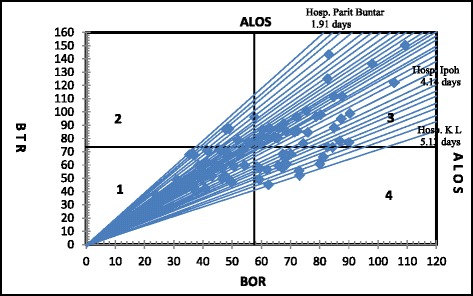
Pabon Lasso graph, public hospitals, Peninsular Malaysia, 2010

Using the values of the BOR and BTR, and their corresponding ALOS, the Pabon Lasso graph for the public hospitals were constructed for years 2006 and 2010 (see Figs. 3 and 4) (Table 3).

**Table 3:** Percentage of all public hospitals in respective performance quadrants, Malaysia, 2006 and 2010

Year	Quadrant 1	Quadrant 2	Quadrant 3	Quadrant 4	Total
2006	27 [31.03 %]	14 [6.09 %]	35 [40.23 %]	11 [12.64 %]	87 [100 %]
2010	37 [42.53 %]	12 [13.79 %]	25 [28.74 %]	13 [14.94 %]	87 [100 %]

Computed from inpatient records, public hospitals, Peninsular Malaysia, 2006 and 2010

Of the 87 hospitals, 27 hospitals (31.0 %) were in quadrant 1 (low BOR, low BTR), 13 hospitals (15.0 %) were in quadrant 2, 36 hospitals (41.4 %) were in quadrant 3 and 11 hospitals (12.6 %) were in quadrant 4 in 2006. Whereas 41.4 % were in quadrant 3, 58.6 % fell outside this zone

Similarly, in 2010, 33 hospitals (37.9 %) were located in quadrant 1, 5 hospitals (5.8 %) in quadrant 2, 31 hospitals (35.6 %) in quadrant, and 18 hospitals (20.7 %) in quadrant 4. Hence, although there has been improvements in the BOR, BTR and ALOS, the share of the public hospitals fell from 41.4 % in 2006 to 35.6 % in 2010.

The descriptive statistics of the public hospitals in Peninsular Malaysia, i.e. the minimum and maximum bed numbers, mean, standard deviation, standard error and the p-values of statistical significance using two-tailed t-test results are presented. An additional Table file shows this in more detail [see Additional file 1].

In 2006, the minimum number of beds was 24 while the maximum was 2331 beds. The minimum and maximum BOR were 20.9 and 97.5 % respectively. Also, the minimum recorded BTR was 12.5 times while the maximum was 132.6 times. As for ALOS, the minimum was 1.9 days and the maximum was 18.2 days.

Similarly, in 2010, the minimum number of beds was 24 with a maximum of 2212 beds. The minimum and maximum BOR was 24.1 and 109.3 % respectively. The minimum recorded BTR was 24.1 times while the maximum was 150.4 times. The minimum ALOS was 1.2 days and maximum of 5.1 days.

## Discussion

Although some improvements have been recorded by public hospitals, the BOR of general, and specialist and non-specialist district hospitals in Malaysia fell significantly below the OECD averages [44], which implies low utilisation rates in Malaysia. The most plausible explanations for the low BOR scores is likely to be a consequence of ineffective planning and the lack of upgrading to expand the facilities at crowded general hospitals, which has resulted in a misallocation of resources. Since private hospitals have expanded only in cities and towns in Malaysia it appears that they have not eased the burden faced by the general hospitals. Interviews show that distance and the quality of provision still matters among inpatients who cannot afford to seek private care.

The BTR of general, and specialist and non-specialist district hospitals in Malaysia was significantly higher than the OECD average [36], which shows excessive turnovers suggesting that it could be a consequence of either discharges resulting from long waiting times, information imperfections between initial and subsequent diagnoses or inadequate services offered to patients. The average BTR of Japan in 2008 was only 15 times [45]. Another explanation found from interviews is the lack of personnel to manage demand [46], particularly because of the migration of medical specialists from public to private hospitals resulting in shortage in the number of medical specialists serving in the public hospital sector.

ALOS of general, and specialist district and non-specialist district hospitals fell from 3.4 days in 2006 to 3.1 days in 2010, which was significantly lower than that of the OECD [43]. The lack of sufficient specialist medical hospitals may be the consequence of the short stay means experienced by the public hospitals. Interviews with specialists at the public hospitals studied showed that a large number of patients prefer private hospitals because the waiting time to carry out services, such as medical resonance imaging (MRI), ultrasound, mammogram and tomogram services was 15 days to 6 months in these hospitals compared to zero days at private hospitals (University of Malaya, 2010)^1^.

Our survey showed that patients who can afford quickly relocate to private hospitals because of the long waiting times in public hospitals. This evidence suggests that private hospitals in Malaysia are not only delivering special care services that are not found in public hospitals, e.g. plastic surgery, extra privacy on certain treatments and comfort during care, such as in Canada, they are also competing with public hospitals to treat similar diseases. Mohideen Kadir of the Consumer Association of Penang and Dato’ Paul Selvaraj of the Federation of Malaysian Consumers Associations reported that even underprivileged poor persons have incurred debt to seek urgent private care in private hospitals because of long waiting times in public hospitals (University of Malaya, 2010). Hence, the expansion in private hospitals may not be the answer for the mis-allocation of services in public hospitals in Malaysia.

## Conclusions

The evidence from this paper substantiates the findings from several other studies that a significant number of public hospitals are inefficient and ineffective [47]. The aggressive restructuring efforts by the Malaysian government that sought to promote the commercialization of health care in the country is not reflected in the performance of public hospitals. This article demonstrates that years after commercialization of health care delivery, inefficiency and poor performance continues to characterize the public health care system in Malaysia. The evidence provided in this study, suggests a mismatch in the allocation and distribution of health care resources in public hospitals. For example, the BOR of some public hospitals in Malaysia is below 25 %, while some recorded over 100 %.

The rapid expansion of the private hospitals has only created a two-tier health care system in which key medical specialists have moved from public to private hospitals [48, 49]. The evidence also shows that the concentration of medical specialists in private hospitals and the under-supply of special services at public hospitals, such as MRI, ultrasound, mammogram and tomogram services has undermined the performance of public hospitals. Hence, the promotion of private hospitals may not be the solution for the burgeoning problems confronting public hospitals. There is a critical need to expand and deepen the provision of specialist care at public hospitals located in cities or to shift specialists care to district hospitals so as to prevent patients in small towns and rural locations from being forced to seek treatment at private hospitals. Private care should largely be focused on complimenting services supplied by public hospitals.

Therefore, this study supports the argument that health care commercialization does not provide the answer to the sub-optimal performance and ineffectiveness of public hospitals [50]. Efforts to enhance efficiency have to be found from within public hospitals, which should start with the reconfiguration of government health care strategies and reducing waste in public hospitals [48]. While this study does not call for the dissolution of the private health care sector, it calls for reforms targeted at public hospitals to lubricate the wheels of its administration [51].

The study has some limitations. Firstly, both the denominators and numerators used to estimate BOR, BTR and ALOS may have measurement problems, and thus, the results must be interpreted with caution. For example, some hospitals may use both certified and uncertified beds within their units as in other health care systems but official reports may comprise only certified beds. As with this study, the BOR, (which is based on midnight bed census at each hospital), are yearly averages, which do not tell us seasonal variations. Also, the data of patients admitted and discharged within the same day of admission are not considered. Nevertheless, the classification of the data used in this study is universal as it is compiled the same way worldwide [41]. Finally, we recommend that new and more effective measurement instruments are devised to offer analysts more reliable tools.

## Additional file

Additional file 1:
**Descriptive statistics, public hospitals, Peninsular Malaysia, 2006 and 2010.** (DOCX 24 kb)

## Footnotes

1 University of Malaya (2010). “Public and Private Hospitals in Malaysia: A Study of Health care Performance”, project financed through E-Science fund, Ministry of Science, Technology and Innovation (MOSTI), Putrajaya, Malaysia.
