# Supplementary material for: Rethinking health care commercialization: evidence from Malaysia
Source: Global Health. 2015 Nov 19;11:44. doi: 10.1186/s12992-015-0131-y (PMC4652362; doi:10.1186/s12992-015-0131-y)
Supplement: Additional file 1: — Descriptive statistics, public hospitals, Peninsular Malaysia, 2006 and 2010. (DOCX 24 kb) [file 12992_2015_131_MOESM1_ESM.docx]

Additional file 1: Table S1: Descriptive statistics, public hospitals, Peninsular Malaysia, 2006 and 2010

| **Hospital capacity** | No. of Hosp. | 87 | 87 |
| --- | --- | --- | --- |
|  | Beds min. | 24 | 24 |
|  | Beds max. | 2331 | 2212 |
|  | Mean | 280.01 | 297.15 |
|  | Std. Dev. | 349.58 | 351.92 |
|  | Std. Error | 37.48 | 37.73 |
|  | P-value | 0.000* | 0.000* |
| **BOR** | Minimum | 20.93 | 24.14 |
|  | Maximum | 97.45 | 109.30 |
|  | Mean | 55.96 | 60.64 |
|  | Std. Dev. | 17.55 | 18.78 |
|  | Std. Error | 1.88 | 2.01 |
|  | P-value | 0.000* | 0.000* |
| **BTR** | Minimum | 12.47 | 36.98 |
|  | Maximum | 132.63 | 150.38 |
|  | Mean | 65.41 | 73.04 |
|  | Std. Dev. | 22.18 | 23.33 |
|  | Std. Error | 2.38 | 2.50 |
|  | P-value | 0.000* | 0.000* |
| **ALOS** | Minimum | 1.89 | 1.91 |
|  | Maximum | 18.16 | 5.13 |
|  | Mean | 3.35 | 3.10 |
|  | Std. Dev. | 1.80 | 0.75 |
|  | Std. Error | 0.19 | 0.80 |
|  | P-value | 0.000* | 0.000* |

Note: Figures in parenthesis refer to p-values, * all means are statistically significant at the 1% level.

Source: computed from inpatient records of all public hospitals in Peninsular Malaysia, 2006 and 2010
